# DISASTER PERCEPTIONS AND PREPAREDNESS BEHAVIORS AMONG U.S. OLDER ADULTS

**DOI:** 10.1093/geroni/igz038.1030

**Published:** 2019-11-08

**Authors:** Melissa Krook, Peter Vitaliano

**Affiliations:** University of Washington, Seattle, Washington, United States

## Abstract

U.S. economic loss from natural disasters hit an all-time high in 2017 with 16 climate events totaling $306 billion. However, disasters’ costliest effects may result from emotional and psychosocial health. Research suggests those who are: seniors, distressed, and/or experience early-life vulnerabilities have increased risk for negative health responses. This study addresses the need to reduce vulnerability/increase preparedness by evaluating how older adults (OA) perceive/prepare for disasters, including influential psychological factors. Literature review results indicate OA are: (1) among our most vulnerable populations for disasters, (2) underprepared, though resources are available, and (3) preparing friends/family before themselves. The Socioemotional Selectivity Theory (SST) posits: alongside aging, time perceptions become constrained, motivations shift, and we prefer positive over negative information. Therefore, I asked: (1) if OA are intuitively resistant to negative information, like impending disasters, how might we reframe it to align with their desire for positive information? (2) If we approach OA through positive experiences, will they be motivated to prepare? I employed a model: preparedness behavior (PB) is a function of vulnerability (V) and resilience (R). A survey was developed to assess how factors of V and R would interact/influence PB. I will pilot test this survey through evaluating community-living OA. PB is expected to be negatively related to V, positively related to R. This study extends disaster research by using psychological variables to predict preparedness and evaluating preparedness motivation using SST as a guiding framework. Results should increase knowledge about OA’s disaster preparedness perceptions and factors to mitigate increased preparedness.

